# Development of Mind–Body Interventions for the Emergency Department

**DOI:** 10.1016/j.acepjo.2026.100404

**Published:** 2026-04-21

**Authors:** Tiffany R. Glynn, DaMarcus Baymon, Kei Ouchi, Timothy B. Erickson, Peter R. Chai

**Affiliations:** 1 Department of Emergency Medicine, Brigham and Women’s Hospital, Boston, Massachusetts, USA; 2 Chemical Biology and Therapeutic Sciences, The Broad Institute, Cambridge, Massachusetts, USA; 3 Department of Psychiatry, Massachusetts General Hospital, Cambridge, Massachusetts, USA; 4 Harvard Medical School, Boston, Massachusetts, USA; 5 Department of Psychosocial Oncology and Palliative Care, Dana Farber Cancer Institute, Boston, Massachusetts, USA; 6 The Koch Institute for Integrated Cancer Research, Massachusetts Institute of Technology, Cambridge, Massachusetts, USA

**Keywords:** mind–body interventions, music, acupuncture, artificial intelligence, distress

## Abstract

Emergency department (ED) visits are a common experience that most individuals will have across their lifetime. ED-based evaluation for acute complaints can be fraught with anxiety and stress due to diagnostic uncertainty, long waiting and boarding times, and the harsh, stimulating environment of the ED. Modulating the physical ED environment can be time consuming, expensive and given regulations, at times nearly impossible. Increased patient stress and anxiety is associated with persistent hypertension, inflammation, and worsening patient experience. Mind-body interventions (MBIs) are nonpharmacologic interventions that leverage the bidirectional relationship between physiological and psychological processes to address universal distress, pain and other commonalities among the ED experience. Many of these interventions like music, mindfulness and acupuncture have been successfully deployed in other clinical settings to mitigate components of stress and improve the patient experience. These interventions have the potential to address the commonalities surrounding ED distress. In this concepts piece we describe MBIs, their potential application in the ED and future directions for research on MBIs in the emergency department.

## 1 Introduction

The emergency department (ED) is a complex, high-acuity clinical environment characterized by prolonged wait times, continuous artificial lighting, noise, and limited privacy—all of which can contribute to significant patient distress. Increasing patient volumes and overburdened hospital capacity have resulted in ED patients facing prolonged wait times, sporadic care in hallways, and, when admitted to the hospital, increased boarding time in the ED. Additionally, acute care models are increasingly shifting to waiting room medicine where triage occurs in parallel with care initiation in order to expedite workup of patients that would normally only start once they are triaged to an emergency room. These structural and environmental effects have strained the US health care system and have profound effects on ED patients including decreased satisfaction, increased stress and frequent and repeated ED visits. Given continued increasing burdens on the health care system and predicted trajectory in patient boarding rates, it is critical that EDs consider novel approaches to address patient distress. One suite of interventions that has yet to be investigated in depth within the ED are low threshold, mind–body interventions (MBIs). MBIs engage the bidirectional relationship between psychological and physiological processes to influence health, emotion, and behavior. Mind–body approaches include cognitive (eg, mindfulness), somatic (eg, tai chi and yoga), sensory (eg, aromatherapy, socially assistive robots), and environmental (eg, light) modulation that have the potential to improve psychological distress and neurophysiologic relaxation. These strategies, also known as complementary and alternative therapies, have the potential to modulate the complex internal and external environment that ED patients inevitably face.

Existing evidence demonstrates the potential for MBIs within the ED. For example, mindfulness-based interventions decrease bodily distress and somatic symptom severity—a common barrier to optimal patient experience and health outcomes.^1^^,^^2^ In a sample of adult inpatients, the majority reported that complementary and alternative medicine approaches would provide relaxation (88%), increase well-being (86%), and increase satisfaction (85%)—reflecting patient beliefs and willingness.^3^ MBIs show acceptability; global systematic reviews indicate an estimated 45% of medical specialists use complementary and alternative medicine (CAM) approaches, and general population estimates range from 24% to 71%.^4^^,^^5^ These data are reflected among a study of 674 ED patients, demonstrating that 74% had used MBI.^6^ Despite the potential for MBIs, emergency medicine–specific data on MBI and CAM use remain limited and an area of important future research.

Mind–body approaches also have the potential to be low threshold, although resource requirements may vary depending on modality. For example, while it may be difficult and costly to redesign patient rooms or increase the physical footprint of the ED, modulation of lighting in individual rooms or providing curated music playlists to address common ED complaints such as pain and anxiety, may represent more accessible strategies that could improve the ED patient experience. MBIs therefore may integrate well within the confines of the ED to address specific environmental inputs that may lead to distress within the patient experience in the ED, although further research will be needed to examine this complex relationship. In this study, we describe several MBIs that have a high potential to be deployed within the ED.

## 2 Specific Modalities of Mind–Body Interventions

### 2.1 Music

Music is a ubiquitous, transcultural experience that almost every human over the course of their lifetime engages with. Listening to desirable or user-selected music can elicit thrills and chills, which correlate with surges of dopamine in the nucleus accumbens and striatum nigra, indicative of euphoria.^7^^,^^8^ Listening to self-selected, favorite music for as short as 10 minutes in length can modulate nociception, resulting in increasing pain tolerance and threshold.^9^ Music is already widely used in outpatient clinics, in the perioperative space, and as a modality for the treatment of pain syndromes, anxiety, and other chronic health conditions. Music as an MBI is easily deployed in an ED setting. Previous groups have used music as an adjunct to generalized pain and as a strategy to address musculoskeletal back pain in the ED.^10^^,^^11^ In these investigations, study teams leveraged patient’s own phones and conducted guided selection of musical playlists and provided headphones to ensure privacy during listening sessions. Other investigations have leveraged centrally managed devices that were loaned to participants during their ED stay.^11^ EDs could consider the deployment of music through curated playlists or leverage music therapy strategies to establish intentions of listening to music and offer options including self-guided song selections or existing generative playlists (eg, streaming music services such as Spotify or Apple Music). For implementation, EDs may also consider stocking disposable headphones or, for individuals without smartphones, loaner devices with preloaded songs or playlists for patients to choose from.

### 2.2 Light

Light is a constant stimulus in the ED. Persistent bright lights are required for clinicians to examine patient and perform critical procedures, yet persistent, sterile white lighting disrupts circadian rhythm, results in immunosuppression, and increases stress. While some EDs seek to mirror circadian patterns by dimming ED lights during evening hours, it is nearly impossible to replicate settings at home where darkness may help restore sleep and circadian rhythm, particularly patients boarding in hallway beds. While modulating lights for the ED as a whole is less feasible, modulating light in individual patient rooms represents a simple MBI that may improve patient experience, restore some semblance of sleep routine, and decrease stress. Modifications of light color and intensity has been shown to decrease circulating cortisol in critically ill patients in the intensive care unit, and modulating light in individual rooms coupled with visual cues of the outside environment may improve orientation in ED patients who are admitted to observation units, are admitted for short stays, or are waiting for test results.^12^, ^13^, ^14^ Although early, these strategies of addressing global health concerns such as orientation, anxiety, and sleep patterns through adjusting lighting conditions should continue to be explored in the ED. Light-based interventions may need to be deployed within rooms given that hallway-based individual lighting may complicate ED care environments. While we do not suggest infrastructure upgrades to existing room lighting, which may lead to significant capital construction costs, there are other potential light-based interventions including battery-powered personal lights or hanging lights that could be used in the ED with minimal infrastructure upgrades.

### 2.3 Acupuncture

Acupuncture is based on the traditional Chinese medicine paradigm that the body has energy flows (*qi*) and that stimulation of *qi* via thin needles at key energy channels (meridians) can restore health. Acupuncture stimulates peripheral sensory nerves leading to release of endogenous opioids such as endorphin and enkephalin, which may explain its mechanism of decreasing pain.^15^ There is a growing body of evidence that acupuncture techniques may be effective in treating common ED complaints such as pain and nausea.^16^^,^^17^ These sessions, ∼15 to 20 minutes in length, have been demonstrated to be feasible and acceptable in ED settings, suggesting that acupuncture may be an unique MBI that could be integrated as an adjunctive therapy for pain and nausea in the ED. Major barriers to deployment of acupuncture in the ED include the need to train staff or employ an acupuncture service in the hospital, and demonstration of efficacy compared with other pain measures. Additionally, research should explore how acupuncture could be used as an adjunctive treatment within the armament of the emergency physician to address pain.

### 2.4 Aromatherapy

Essential oils are widely used for anxiolysis and stress reduction. Linalool, the active component of lavender binds the γ-aminobutyric acid (GABA) receptor and induces vasodilation, resulting in anxiolysis and decreased blood pressure. Menthol, the active component of peppermint, binds gastric calcium channels and the family of transient receptor potential channels, resulting in gastric smooth muscle relaxation, thereby decreasing nausea, spasms, and pain. Finally, inhaled isopropyl alcohol when used as aromatherapy has recently been demonstrated to be as effective as ondansetron in addressing nausea in ED patients, suggesting that using different aromas could serve as an adjunctive treatment for common ED complaints.^18^ While these investigations are promising, larger-scale, reproducible trials that enroll ED patients, standardize dosing and delivery of aromatherapy, and facilitate delivery in ED settings are needed to advance this potential MBI. Multiple strategies exist to deliver aromatherapy in the ED including the use of diffusers and aromatherapy inhalation tubes, whicht can deliver a reliable dose of aromatherapy to patients without allowing aroma to diffuse outside of the patient’s surroundings or other patient rooms. These supplies are relatively low cost and can be assembled by ED staff, but use of essential oils in aromatherapy may need hospital approval or, when used in research, regulation under the US Food and Drug Administration (FDA).

### 2.5 Socially Assistive Robots

Social connection and affiliative touch are core regulators of physiological stress responses. Interactive companions, such as artificial intelligence (AI)-enabled robotic pets, capitalize on this mind–body pathway by providing tactile comfort and socially responsive feedback. Soft, fur-like textures activate cutaneous mechanoreceptors associated with soothing parasympathetic activity, while contingent, affective responses engage neural circuits implicated in attachment and emotion regulation, including the oxytocinergic and vagal systems. Data suggest that haptic and social interaction with responsive devices can attenuate stress-related autonomic arousal and promote feelings of safety. Evidence from the robotic seal PARO, an FDA-approved, neurotherapeutic device, suggests that these interventions positively impact arterial pressure, pulse, respiration, oxygenation, stress, anxiety, and pain levels among hospitalized patients.^19^ The most recent and comprehensive 2023 systematic review and meta-analysis of 12 RCTs (1461 participants) found that PARO had a moderate effect on reducing medication use and small effects on anxiety, agitation, and depression in older adults with dementia.^20^ These rapidly deployable robots could in the future be deployed in the ED to address acute delirium, address anxiety of novel surroundings commonly experienced by individuals with dementia, or provide stress relief for patients boarding in EDs. These potential uses of socially assistive robots will need to be balanced against the high relative cost of these systems, strategies for sterilization in between use, potential interference in robots that use wireless telecommunication to achieve tasks, and durability in the ED.

### 2.6 Restorative Nourishment

Nutritional intake influences mood and stress physiology through multiple metabolic-interoceptive pathways, including glucose regulation, gut–brain signaling, and serotonergic activity. Acute food deprivation and hypoglycemia (a consequence of long wait times in the ED) increase sympathetic activation and irritability (also known as hangry), whereas nutrient consumption restores homeostasis and supports parasympathetic recovery. Evidence from nutritional psychiatry demonstrates that consumption of snacks, particularly those rich in complex carbohydrates, antioxidants, and vitamins, correlates with lower levels of anxiety and emotional distress.^21^^,^^22^ Within the ED, a small prospective study demonstrated distribution of popsicles to eligible pediatric ED patients resulted in increased parent satisfaction scores post visit.^23^ In the ED context, nourishment represents a plausible, low-cost adjunctive strategy to address physiologic and affective consequences of prolonged fasting for appropriate patients. Operational constraints including nothing-by-mouth status for patients being evaluated for acute complaints may limit the use of nutritional offerings. Despite this, patients with prolonged wait times and those boarding in the ED may be prime targets for nutritional interventions that may impact their experience. Deployment of different nutritional offerings will need to balance palatability with nutritional content, potential food allergies, and other dietary restriction of the ED patient population.^24^

## 3 Conclusion

While there is promise for the use of MBI within the ED, the evidence base for most modalities remains preliminary and ED-specific data are limited. Despite this, preliminary work suggests that these strategies could be effective, adjunctive tools in the ED and warrant exploration, especially in the specific environments of the ED. The National Center for Complementary and Integrative Health provides a structured conceptual model and competitive funding structure that can support feasibility, acceptability, and, eventually, effectiveness and reproducibility of these MBIs. We urge emergency medicine researchers and practitioners of MBIs to consider translation of MBIs into the ED setting to modulate overall stress, pain, anxiety, or other conditions that may contribute to the overall ED experience.

## Author Contributions

PRC was responsible for conception of the manuscript, acquisition of funding, and writing the initial draft with TRG. TRG was responsible for completion of the first draft and critical revisions of the manuscripts. DEB, KO, and TBE were responsible for critical revisions and conception of the manuscript.

## Financial Support

PRC was funded by 10.13039/100000002National Institutes of Health (NIH) DP2DA056107; TRG was funded by NIH K23DA060719 and the Kahn Award from the Department of Emergency Medicine, Brigham and Women’s Hospital. PRC and TRG were funded by NIH R34AT013522.

## Conflict of Interest

PRC and TBE hold equity in Biobot Analytics a small business that develops wastewater-based analytics for substance use. PRC is a consultant for Syntis Bio and ABSCOTx.
